# Nursing Students’ Retention of Knowledge by Basic Knowledge Type: An Exploratory Study

**DOI:** 10.3390/ijerph19095461

**Published:** 2022-04-29

**Authors:** Hiromi Kawasaki, Satoko Yamasaki, Susumu Fukita, Mika Iwasa, Tomoko Iki

**Affiliations:** 1 Division of Nursing Sciences, Graduate School of Biomedical and Health Sciences, Hiroshima University, Hiroshima 734-8551, Japan; morisato@hiroshima-u.ac.jp; 2 School of Nursing, Dokkyo Medical University, Tochigi 321-0293, Japan; s-fukita@dokkyomed.ac.jp; 3 Faculty of Nursing, Shitennoji University, Habikino 583-8501, Japan; m-iwasa@shitennoji.ac.jp; 4 Graduate School of Nursing, Kansai University of Nursing and Health Sciences, Awaji 656-2131, Japan; t.iki@kki.ac.jp

**Keywords:** nursing students, environment knowledge, learning

## Abstract

Students’ interests help determine their learning effectiveness and knowledge acquisition and retention. It is necessary to confirm whether there is a difference in the way in which content being learned is remembered by the content type. In this study, we examined the characteristics of nursing students’ retention of physiological knowledge and environmental knowledge by utilizing scores obtained in class. The participants comprised 57 nursing students who had taken a class twice—once in their second year and once in their third year. Before and after each class, students completed an 11-question survey with human health and comfort items based on nursing core competencies and Sphere standards. The correct answer rate was calculated using a logistic regression model to account for inter- and intra-individual variations. The estimated correct answer rate per individual showed one of three trends: (1) increasing and decreasing depending on the lesson topic (knowledge type), (2) increasing overall after decreasing, and (3) increasing gradually. Physiological knowledge was retained well, whereas knowledge pertaining to the environment was retained poorly. Even with knowledge of the environment, the knowledge that students apply to their daily lives and social events was maintained.

## 1. Introduction

Nurses must be prepared for various roles in diverse settings. Although many people assume that nurses work only in hospitals [1], nurses’ duties go extend from tending to patients and supporting treatment in hospitals to helping people in the community maintain and improve their daily health [2]. In the future, the expectation that they can provide knowledge and care related to health and wellbeing outside hospital settings may increase [3]. All nurses must possess basic knowledge on how to support people’s routines and be able to apply it [4]. Indeed, applying theoretical knowledge (for example, patient assessment) to professional nursing practice is a major component of nursing competency [5,6]. Moreover, in a disaster or emergency, all nurses must swiftly apply their expertise, even outside the hospital.

As healthcare becomes more sophisticated, nursing skills are becoming increasingly specialized. In particular, in the context of hospital work, nurses trained to handle advanced medical technologies competently are critical. A person’s motivation to learn depends on their interests and on the state of their internal and external environment. With intrinsic motivation to learn, students take deeper interest and can continue learning even when facing difficulties [7,8]. Learners tend to be more motivated to learn material directly relevant to them [9,10] or to their chosen profession and qualifications [11]. Many nursing students aspire to be nurses who are competent in advanced medical technology [12]. Given the importance of basic nursing skills for nursing students, it is crucial to ensure the acquisition and retention of these skills without causing boredom in students [13,14].

The purpose of this study is to clarify whether nursing students remember nursing knowledge differently by knowledge type. The results clarify what knowledge nursing students do not remember easily, yielding the opportunity to examine improvement methods.

## 2. Materials and Methods

### 2.1. Study Design

This exploratory study was conducted using a questionnaire administered to nursing students before and after they attended two classes held in June 2019 and June 2020, in the students’ second and third years, respectively. The classes had different goals but required the same knowledge. Students’ questionnaire scores were analyzed; each questionnaire consisted of 11 items, with one point awarded for each correct answer.

### 2.2. Recruitment

Students who had completed two specific classes within the nursing college curriculum were targeted for recruitment. All students enrolled in 2018 were eligible. The first class was a health management class in their second year, conducted in June 2019. The second was a community health class in their third year, in June 2020. Students who took both classes were selected. Sixty students met the criteria, 57 of which agreed to have their scores analyzed in this study.

### 2.3. Data Collection

**Figure 1:**
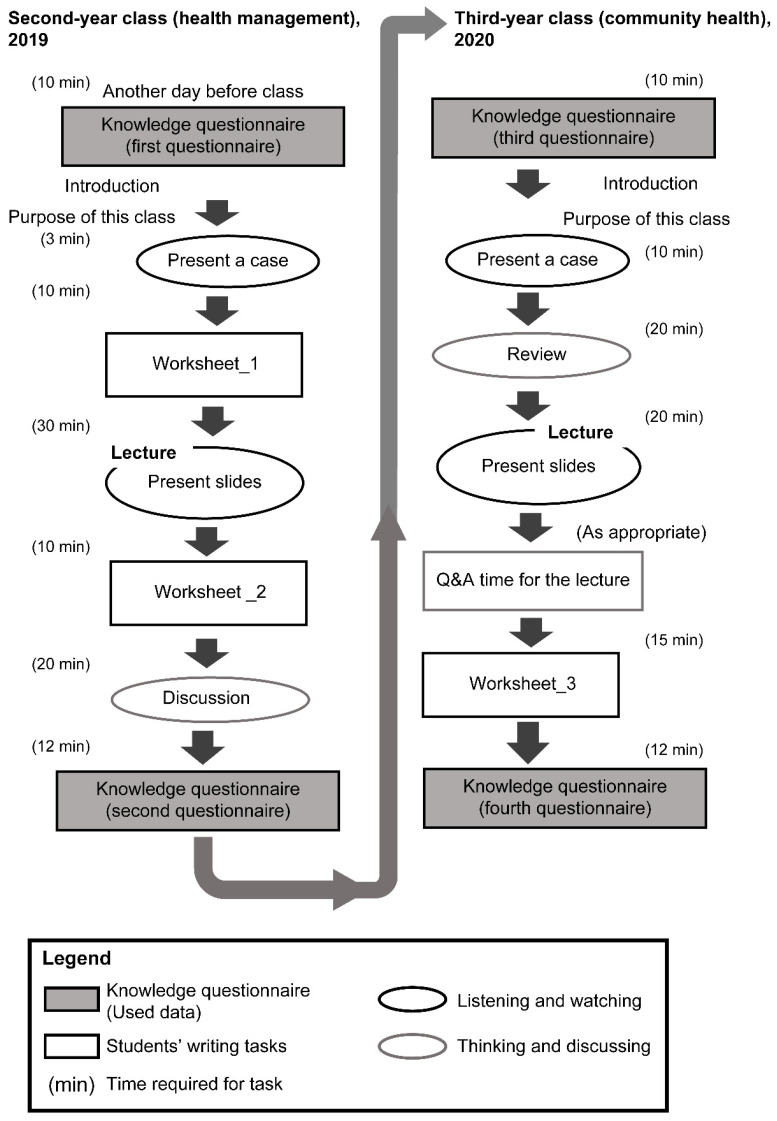
The flow of two classes over an interval of one year. The white boxes indicate students’ writing tasks. The gray boxes represent the analyzed data. The time required for each task is shown in parentheses.

The respective courses had different attainment goals but utilized the same knowledge. Students repeatedly responded to 11 key biomedical questions based on core competencies included in the Japanese basic nursing curriculum [2] and the Sphere standards [15]. Knowledge was assessed using 11 items on standard reference values (Table 1). The questionnaire was administered before and after the second- and third-year classes. A total of four observations were obtained. Figure 1 provides an overview of the study design, including the content of the two classes attended by the students in their second and third years.

### 2.4. Class Contents

#### 2.4.1. Second-Year Class (Health Management) Contents

The learning unit used for the research included in the subject of Health Management was “Health management at the evacuation center.” The class objective was to consider healthcare in evacuation shelters in times of disaster. The required knowledge was assessed using 11 items selected from the core competencies included in the Japanese primary nursing curriculum [2] and the Sphere standards [15].

The items were selected from the core competencies adopted as the class goals and foundational knowledge. They included knowledge of the mechanisms underlying mental and physical health, ability to assess individuals in the context of their daily activities, knowledge of basic human needs and self-care, and the ability to incorporate an individual’s surrounding environment when making an assessment.

The objectives were as follows:

1. To understand the environmental conditions necessary for a normal life;
2. To develop an interest in assessments involving an individual’s environment;
3. To be able to suggest ways to prevent health issues.

The appropriate levels of interpersonal distance, noise, indoor brightness, temperature, humidity, water intake, and average urination per day were taught in class. The students were then asked to complete worksheets to assess environmental conditions. Based on the worksheets, students discussed healthcare provision in evacuation shelters, the risks involved in this setting, and ways to preserve evacuees’ health.

Hiroshima Prefecture, where the participating university is situated, frequently experiences landslides and heavy rains. A disaster caused by heavy rain was the subject of this class’s lecture components. Students were presented with the scenario described below.

*One day in June, while the students were attending a lecture, an evacuation order was issued by Hiroshima City at 15:00 because heavy rains had caused the river near the university to swell above dangerous levels (bank-full stage). Residents had already evacuated to the university’s gymnasiums. The students evacuated to the gymnasium of a junior high school neighboring the university, where each person was given a bottle of mineral water (500 mL) and a blanket. The windows could not be opened because of heavy rains, and the lights went out due to the power outage. The shelter temperature/humidity was 28 °C/90%.*

#### 2.4.2. Third-Year Class (Community Health) Contents

The same disaster scenario presented in the second-year class was utilized again as a case study in community health care. Here, the participants learned about the characteristics of different communities and residents that were made to evacuate during the lecture. Students then assessed the health risks posed to the evacuated residents, discussed ways to prevent them, and developed corresponding nursing plans. Third-year nursing students needed to deepen their competence in the action, especially to the extent that they can relate the actual provision of healthcare to others.

During class, students identified situations in need of improvement based on the results of the assessment. Then, they considered measures aimed at improving these situations and modifications to realize these measures. The third-year students responded on the same 11 knowledge items as the second-year students. The person in charge decided whether the lesson goals were achieved by using the description in the worksheets.

The central goals were to understand how local governments manage health crises and emergency-shelter operations related to evacuees’ lives and health following a disaster. The specific goals were:

1. To understand different forms of health crisis management (HCM) and their associated conditions;
2. To be interested in the systems used in HCM;
3. To think about the different ways in which disaster-related damage can be addressed by HCM.

The third-year class consisted of a series about the lifestyles and health of community residents. The students reviewed the work they had carried out the previous year, confirmed the fundamental reference values, and then learned information in the context of the same case study. Students were assessed by reviews and reports to achieve their goals. This information included:

- Designing initial crisis-response systems: (i) ascertaining the situation, (ii) helping residents make informed decisions, and (iii) participating in relief efforts;
- Explaining the criteria for someone in urgent need of care and ways to treat or manage them;
- Developing strategies to enhance residents’ abilities to help themselves and others;
- Taking systematic approaches to health management for employees in disaster-stricken areas;
- Designing systems for continually adjusting areas where support and dispatched personnel are stationed.

### 2.5. Analysis Method

We estimated the correct answer rate and magnitude of changes across each of the four questionnaires. Generally, the percentage of questions a test-taker answers correctly (correct answer rate) will vary according to other test-takers’ scores on the same test on different dates [16,17]. Students’ questionnaire performance was corrected to account for such inter- and intra-individual variation instead of analyzing raw questionnaire scores. This quantity—the estimated correct answer rate (eCAR)—was derived using the method described below. The answers to each question were judged as correct or incorrect. A correct answer was given one point and an incorrect answer was given zero. Since the answer is binary (correct or incorrect), a logistic regression model was applied to estimate the correct answer rate on the four questionnaires.

Let $p_{j}(Y=1|t)$ be the correct answer rate of question *j* for each questionnaire *t*(j=1,⋯,11:t=first,⋯,fourth), where *Y* denotes a stochastic binary variable defined by $Y=\{\begin{array}{c} 1\;\left(\mathrm{Correct}\right)\;\\ 0\;\left(\mathrm{Incorrect}\right). \end{array}$

We assume the following logistic model:

(1) $$log\frac{p_{j}(Y=1|t)}{1-p_{j}(Y=1|t)}=\mu_{j}+\delta_{1j}d_{1}+\delta_{2j}d_{2}+\delta_{3j}d_{3}$$

where $d_{1},\;d_{2}\;\mathrm{and}\;d_{3}$ are dummy variables shown as follows:

$$d_{1}=\{\begin{array}{l} 0\left(t=1\right)\\ 1\left(t\geq 2\right),\; \end{array}d_{2}=\{\begin{array}{l} 0\left(t\leq 2\right)\\ 1\left(t\geq 3\right),\; \end{array}d_{3}=\{\begin{array}{l} 0\left(t\leq 3\right)\\ 1\left(t=4\right). \end{array}$$

**Figure 2:**
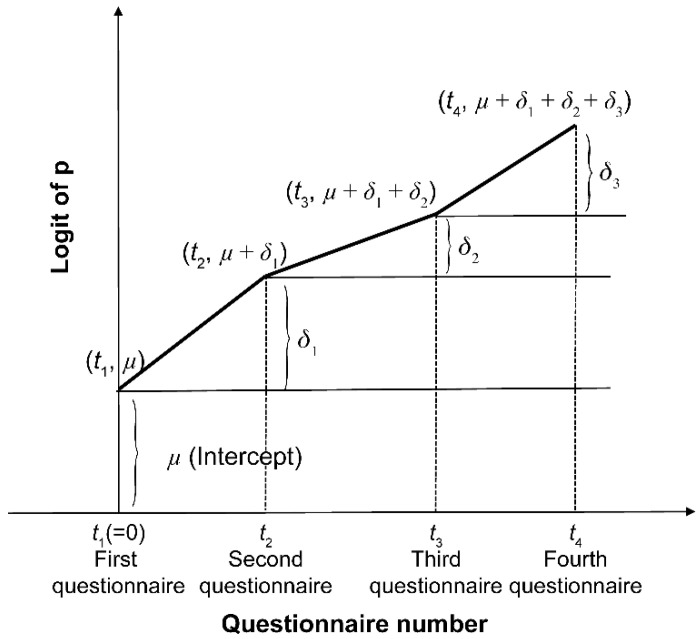
The estimated correct answer rate of the four questionnaires *δ*. The intercept is the estimated correct answer rate for one observation. From Time 2 onward, the graph was drawn by adding the estimated correct answer rates.

The model (1) is schematically displayed in Figure 2.

We visualized the magnitude of changes across the four questionnaires. For this, the difference in the correct answer rate for each question (*j* = 1, …, 11) across the four questionnaires (*t* = first questionnaire, …, fourth questionnaire) and the eCAR was graphed. The method used to create the graph is shown in Figure 2.

To clarify, *t* “first questionnaire,” “second questionnaire,” “third questionnaire,” and “fourth questionnaire” refer to the questionnaire taken before the second-year class, after the second-year class, before the third-year class, and after the third-year class, respectively (Figure 1). R version 3.6.2 [18] was used to conduct this analysis.

### 2.6. Ethical Considerations

The study’s purpose, methodology, and how their data would be handled was explained to the students. They were informed that participation was voluntary and that they retained the right to refuse participation or withdraw from the study. The participants provided informed consent in writing and only their data were analyzed. All data were de-identified by assigning them with identification numbers unrelated to the corresponding participant.

## 3. Results

The estimated parameters ${\hat{\delta }}_{1j},\;{\hat{\delta }}_{2j},\;{\hat{\delta }}_{3j}$ for each question *j* are listed in Table 2. Changes in the eCAR for each question can be classified into three patterns:

1. Pattern #1 (the item’s eCAR was higher on the second questionnaire than on the first);
2. Pattern #2 (the item’s eCAR was lower in the second questionnaire than the first but higher in the third, a year later);
3. Pattern #3 (the item’s eCAR was minimally variable across the four assessment points).

### 3.1. Pattern #1: eCAR Increased after the First Class (Second > First)

**Figure 3:**
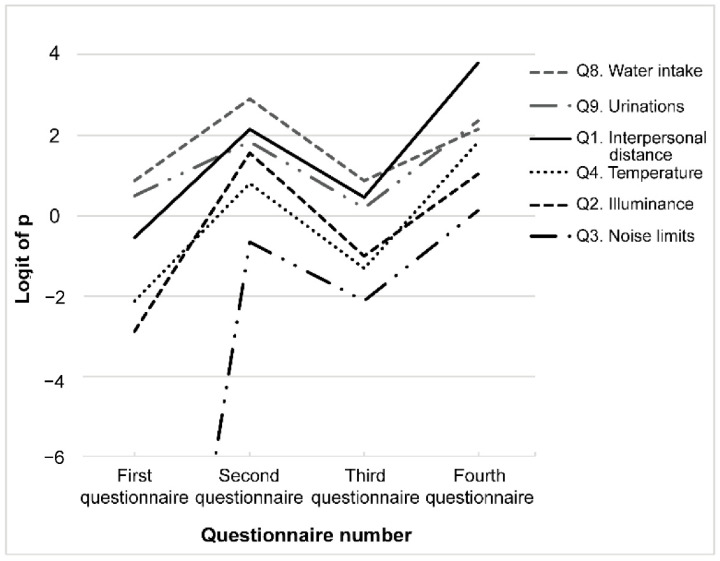
Pattern #1: Correct answer rate improves after the first class. Questions with an estimated correct answer rate of “second > first” were selected. Each question is indicated using a different line.

Changes in the eCAR for the six questions wherein the rate increased after the second questionnaire are shown in Figure 3. In the first questionnaire, students’ eCAR was the highest for required water intake (Q8), followed by daily urination (Q9), interpersonal distance (Q1), indoor temperature (Q4), illuminance (Q2), and community noise limits (Q3). Students’ eCAR for these items on the second questionnaire were significantly higher than that on the first questionnaire. The performance decreased significantly on the third questionnaire conducted a year later. Nevertheless, after the class (fourth questionnaire), their eCAR significantly improved for Q1, Q9, Q8, Q4, Q2, and Q3 (Figure 3).

### 3.2. Pattern #2: eCAR Decreased after the First Class but Increased a Year Later (Third > Second < First)

**Figure 4:**
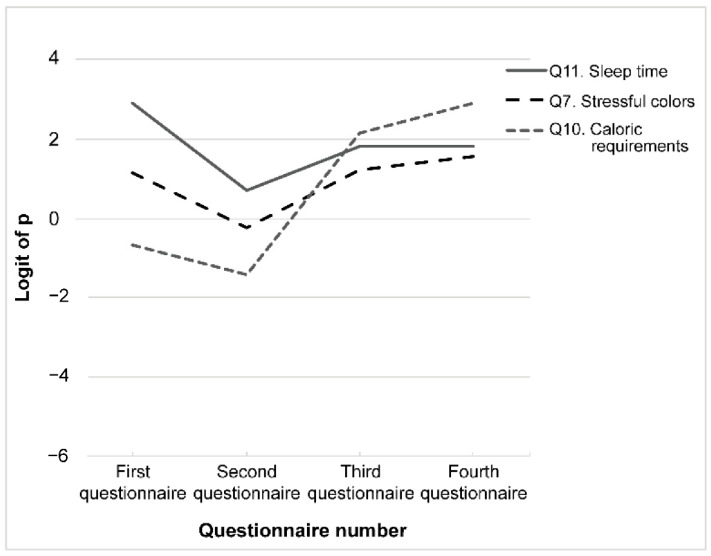
Pattern #2: Correct answer rate decreased after the first class. Questions with an estimated correct answer rate of “third > second < first” were selected. Each question is indicated using a different line.

In the first questionnaire, students’ eCAR was the highest for recommended sleep time (Q11), followed by stressful colors (Q7) and caloric requirements (Q10). In the second questionnaire, the retention rates were significantly lower for Q11 and Q7. One year later, students performed significantly better on Q11, Q7, and Q10 in the preclass (third) questionnaire than in the previous (second) assessment and performed roughly the same on the preclass and postclass (fourth) questionnaires (Figure 4).

### 3.3. Pattern #3: Minimal Variation over Time

**Figure 5:**
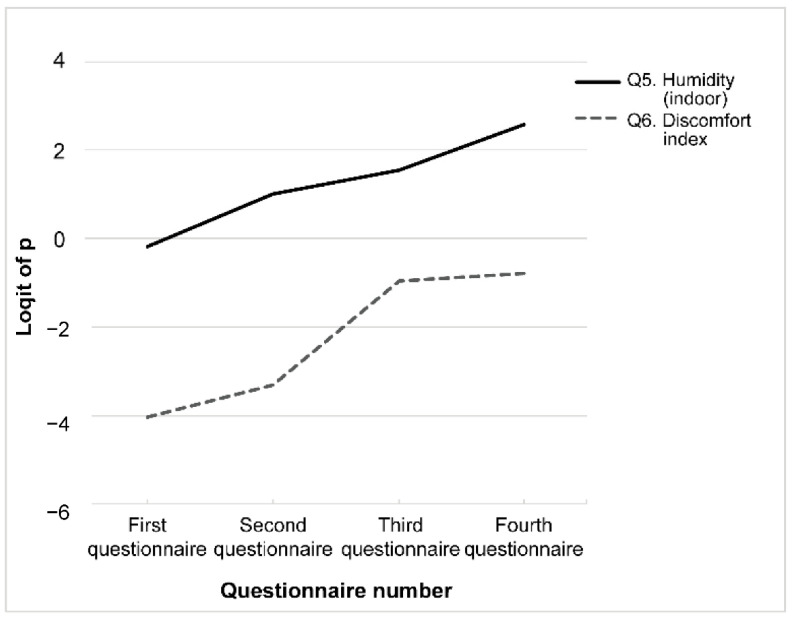
Pattern #3: Minimal variation over time. Questions with an estimated correct answer rate of minimal variation were selected. Each question is indicated using a different line.

Students’ eCAR on questions about indoor humidity (Q5) and discomfort index (Q6) were generally lower on the first questionnaire. Humidity was recalled significantly better on the postclass (second) questionnaire, but its retention remained stable in all the subsequent questionnaires. The discomfort index was recalled significantly better on the preclass (third) questionnaire a year later, but no better (or worse) than on the final (fourth) questionnaire (Figure 5).

## 4. Discussion

The purpose of this study was to clarify whether knowledge is remembered differently depending on knowledge type. Questionnaires based on 11 knowledge items selected from core competencies [2] were used. The 11 items consisted of physiological and environmental information. The scores on the questionnaire—repeated four times—were calculated using a logistic model to determine the eCAR to consider intra- and inter-individual variability for each learned characteristic. The traits were divided into three groups.

The first group’s estimated response rate fluctuated with each lesson, including the most remembered content: interpersonal distance. The estimated response rate recovered for the second questionnaire and increased for the third. Six of the knowledge items followed the same pattern. Students’ correct answer rate improved after the first class, declined a year later, and increased again when the material was reintroduced in the second class. The effectiveness of these six facts, evaluated using eCAR, has a classic shape of a forgetting curve [19].

Daily water intake and urination were among the most accurately remembered items by students before the second-year (first questionnaire) and third-year classes (third questionnaire). Because of their direct relevance to physiological knowledge and practice value, these quantities were probably retained particularly well by students [9]. After the water intake (Q8) and urination (Q9) items, students had the best recall for the interpersonal distance (Q1) item before the first class (first questionnaire). However, by the final assessment (fourth questionnaire), this question had the highest eCAR. One possible explanation why this quantity was not recalled as accurately on the first questionnaire could be that personal space is not directly relevant to physiological knowledge. Moreover, we suspect that its high recall is better attributed to social conditions. The COVID-19 pandemic had already spread in Japan in June 2020. By then, students had been inundated with media reports on social distancing that propagated the notion that interpersonal distance can become a matter of life or death, which might explain why the recall of interpersonal distance has increased. Illuminance, noise, and temperature are all environmental conditions important to human health. However, students’ eCARs on the related questions before the first class (first questionnaire) were lower than the reference quantities directly implicated for survival. One possible explanation for this difference could be that since students envisioned their workplace as a regulated hospital environment, they determined little value in its practice [9]. The nursing college curriculum devotes considerable time to recovery and rehabilitation. This is because a nurse’s central duty is to help people recover from mental or physical illnesses. Substantial nursing education research has been devoted to barriers that prevent students from practicing physical assessments [20]. Studies have found that the task is perceived as complex and involving many obstacles. Environmental assessment and modification have major significance for home-based nurses who tend to and care for patients with chronic diseases after their return [5]. For example, the knowledge that respiratory disease in older adults has been linked to cumulative indoor temperature exposure [21] is important to nursing students. Thus, despite the close association between the environment and health, they may fall out of the practice of conducting physical assessments.

Knowledge retention is not static; it fluctuates with time [19]. Therefore, nursing students are repeatedly tested on biological reference values related to survival in upper-level coursework and clinical practicum. Nursing instructors should remind their students to take special care in memorizing reference values for environmental conditions, which indirectly maintain human health, although they are mentioned less frequently than physical or physiological indicators in upper-level classes. For example, students aiming to become school health teachers must actively memorize information relevant to supporting children’s exercise and learning environments. Similarly, those aiming to become public health nurses should focus on knowledge specific to occupational environments and home-based care. Basic knowledge of reference values for environmental conditions is crucial for operating in evacuation shelters or home healthcare. Regardless of their future direction, it is imperative to verify whether students retain the knowledge they gain. The knowledge that students determine to be of high value is generally retained [9]. The knowledge related to COVID-19 was maintained better than other knowledge, suggesting students considered it to be of greater value. Knowledge of the environment, such as room temperature, illuminance, and noise must be deliberately taught to help students see that it is valuable.

Further, for the group with decreased eCAR after the class, student performance on recommended sleep time, stressful colors, and caloric requirements before the first class (first questionnaire) was not more damaging than others. Reference values for each were presented in class. However, since they were unrelated to the emergency evacuation measures in the case study, these were not covered in the postclass assessment. Since these facts were unrelated to the tasks assigned, the recall decreased after the class. This decline is likely because students judged them as not worth applying [9]. Knowledge generally declines over time [19]; however, it did not decrease after a year in this group. The third questionnaire on sleep time, tense colors, and required calories may have been influenced by life and learning in other classes. We believe that the knowledge represented by these three factors should be learned by students in their first year.

Finally, the group whose eCAR gradually increased comprised two questions. In the first questionnaire, students showed poor knowledge of the threshold value of the discomfort index (that is, the temperature–humidity index). They did not perform significantly worse on this item after the second-year class (second questionnaire), presumably because the discomfort index and its reference scale were included in the lecture material. If knowledge is maintained after six months, its relevance could be supported [22]. The eCAR for the humidity and discomfort index increased during the third questionnaire, which was conducted a year after the second questionnaire, demonstrated the relevance of the knowledge related to these indices. Because of the continuous learning during the year or due to the spread of COVID-19 in 2020, information regarding the medical environment and prevention of heatstroke by wearing masks was reported daily. The university also alerted students to recite this information. This indicates that it has been repeatedly learned owing to accidental social events. In the absence of the COVID-19 pandemic, this group’s response may have been different. The studied and utilized information in the intervening year could explain this pattern. This suggests that knowledge closely intertwined with students’ daily lives may be readily retained, even if only presented once in their second year.

We attempted to highlight the characteristics of the learning material by considering individual variations in questionnaires and between students. Other appropriate analytical methods were also considered. Estimating the correct answer rate using logistic regression analysis helped in adjusting for intrapersonal and interpersonal variability and reveal the retention characteristics of a particular population. The results of the study suggest that this method can be used to detect changes in and characteristics of content scores over time, not only for nursing students but also for other populations. The results in this study may be different from those of previous studies because this study coincided with the spread of COVID-19. In previous studies, learning material had profession-related learning characteristics [23]. For nursing students, the learning traits were a great interest in knowledge of a patient’s physical condition and related needs. Difficulties in learning have been treated as individual characteristics by past researchers [24]. However, in this study, such difficulties were not only the characteristics of an individual. As a result of considering the fluctuations within and between individuals, it was objectively shown that nursing students as a whole possess characteristics related to knowledge acquisition. Nursing students need to consciously strengthen their knowledge of a patient’s environment and related needs, and educational plans should be created to consider this.

### Limitations

This study was a longitudinal comparison of a limited subset of nursing students based on just two classes (one year apart), during the ongoing COVID-19 pandemic. Questionnaire items that were created in the class had been reused. However, the questionnaire items covered only limited areas of learning; therefore, other areas of learning, i.e., physiological knowledge, environmental knowledge, anatomy, politics, and economics also need to be investigated. How other nursing students learn and adapt in the aftermath of different significant events in society needs to be investigated in future studies. It is necessary to observe the areas in which students work; that is, whether the characteristics of the content change in the advanced year.

## 5. Conclusions

This study clarified the characteristics of knowledge retention among nursing students by visualizing their performance on a knowledge assessment over time. Their scores were adjusted using logistical models to account for differences between individuals and individual fluctuations. This adjustment method suggests that it is possible to consider the characteristics of students’ vocational orientation.

We found that physiological knowledge was retained well, whereas knowledge about topics such as environment, not directly related to the students’ practice, was retained poorly. Nurses’ responsibilities are no longer limited to routine physical care and helping patients recover from illness in healthcare facilities, and nursing education must pay special attention to ensuring that students acquire knowledge that is not directly related to illness recovery (e.g., environment), yet bears high significance in sustaining patients’ health and well-being. As the body of information needed to conduct modern medical care grows, educational designs that allow students to use the same knowledge in different situations can save students’ time in gathering knowledge and may lead them to advanced thinking.

## Figures and Tables

**Table 1:** Knowledge questionnaire.

Category	Question ID (Shorthand)	Question [Unit]
Reference values: Interpersonal environment	Q1 (interpersonal distance)	How much distance should be maintained in interpersonal interactions (in public or social settings)? [m]
	Q2 (illuminance)	How bright should patients’ rooms be in a hospital ward? Please give respective values for daytime and nighttime in lux. [lx]
	Q3 (noise limits)	What are the reference levels for community noise in the daytime and nighttime? Please give each value in decibels. [dB]
	Q4 (temperature)	What temperature should be maintained indoors in the summer ensure comfort? [°C]
	Q5 (humidity)	What humidity should be maintained indoors in the summer ensure comfort? [%]
Reference values: Physiological/internal	Q6 (discomfort index)	In terms of discomfort index, above what value is perceived as uncomfortable?
	Q7 (stressful colors)	Which colors provoke stress?
	Q8 (water intake)	How much water does a person need per day? [mL/d]
	Q9 (urinations)	How many times does a person urinate per day? [times/d]
	Q10 (caloric requirements)	How many calories does a person need per day, that is, what is their Estimated Energy Requirement? Write the value for a young adult male or female in their 20s of (physical) activity level II. [kcal/d]
	Q11 (sleep time)	How many hours should a person sleep to stay healthy? [h/d]

**Table 2:** Parameter estimates for each question.

Reference Values	Question	Parameter	Estimate	Standard Error	*z* Value
Interpersonal environment	Q1. Interpersonal distance	(Intercept)	−1.030	0.301	−3.423
		δ1	2.704	0.472	5.733 ***
		δ2	−1.709	0.450	−3.801 ***
		δ3	3.349	0.767	4.366 ***
	Q2. Illuminance	(Intercept)	−2.890	0.593	−4.873
		δ1	4.438	0.688	6.452 ***
		δ2	−2.577	0.460	−5.601 ***
		δ3	2.059	0.425	4.841 ***
	Q3. Noise limits	(Intercept)	−18.566	863.945	−0.021
		δ1	17.879	863.945	0.021
		δ2	−1.447	0.515	−2.810 **
		δ3	2.245	0.507	4.432 ***
	Q4. Temperature	(Intercept)	−2.140	0.432	−4.959
		δ1	2.913	0.517	5.633 ***
		δ2	−2.095	0.432	−4.848 ***
		δ3	3.134	0.501	6.256 ***
	Q5. Humidity	(Intercept)	−0.176	0.266	−0.601
		δ1	1.206	0.402	3.003 **
		δ2	0.518	0.460	1.126
		δ3	1.036	0.625	1.659
Physiological /Internal	Q6. Discomfort index	(Intercept)	−4.035	1.009	−3.990
		δ1	0.711	1.239	0.574
		δ2	2.373	0.778	3.051 ***
		δ3	0.168	0.410	0.409
	Q7. Stressful colors	(Intercept)	1.122	0.308	3.647
		δ1	−1.369	0.407	−3.361 ***
		δ2	1.466	0.413	3.546 ***
		δ3	0.328	0.470	0.699
	Q8. Water intake	(Intercept)	0.856	0.290	2.955
		δ1	2.035	0.660	3.083 **
		δ2	−2.035	0.660	−3.083 **
		δ3	1.284	0.520	2.471 *
	Q9. Urination	(Intercept)	0.464	0.272	1.707
		δ1	1.348	0.468	2.878 **
		δ2	−1.637	0.465	−3.520 ***
		δ3	2.166	0.538	4.023 ***
	Q10. Caloric requirements	(Intercept)	−0.693	0.281	−2.467
		δ1	−0.738	0.438	−1.685
		δ2	3.571	0.547	6.531 ***
		δ3	0.750	0.734	1.023
	Q11. Sleep time	(Intercept)	2.890	0.593	4.873
		δ1	−2.197	0.656	−3.348 ***
		δ2	1.119	0.474	2.363 *
		δ3	3.440 × 10^−16^	0.539	0.000

***: *p* < 0.001, **: *p* < 0.01, *: *p* < 0.05.

## Data Availability

The datasets used and analyzed during the current study are available from the corresponding author on reasonable request.

## References

[1] Hoeve Y.T., Jansen G., Roodbol P. (2014). The nursing profession: Public image, self-concept and professional identity. J. Adv. Nurs..

[2] Japanese Nursing Association (2016). Nursing Clinical Ladder. Tokyo: Japanese Nursing Association. https://www.nurse.or.jp/home/publication/pdf/fukyukeihatsu/jissen.pdf.

[3] McCollum M., Kovner C.T., Ojemeni M.T., Brewer C., Cohen S. (2017). Nurses Improve Their Communities’ Health Where They Live, Learn, Work, and Play. Policy Politics Nurs. Pract..

[4] Logrippo M.T., Brienza-Arcilla D., Raoji N.V., Polakowski J. (2020). A call to act: RN volunteers needed in their communities. Public Health Nurs..

[5] Fukada M. (2018). Nursing Competency: Definition, Structure and Development. Yonago Acta Med..

[6] Bergman E.M., De Bruin A.B., Herrler A., Verheijen I.W., Scherpbier A.J., Van Der Vleuten C.P. (2013). Students’ perceptions of anatomy across the undergraduate problem-based learning medical curriculum: A phenomenographical study. BMC Med. Educ..

[7] Ferreira M., Cardoso A.P., Abrantes J.L. (2011). Motivation and Relationship of the Student with the School as Factors Involved in the Perceived Learning. Procedia Soc. Behav. Sci..

[8] Aguilera-Hermida A.P. (2020). College students’ use and acceptance of emergency online learning due to COVID-19. Int. J. Educ. Res. Open.

[9] Keller J.M. (1987). Development and use of the ARCS model of instructional design. J. Instr. Dev..

[10] Ho Y.Y., Lim W.Y.R., Sanger C.S., Gleason N.W. (2020). Educating Adult Learners: Bridging Learners’ Characteristics and the Learning Sciences. Diversity and Inclusion in Global Higher Education.

[11] Paul J., Jefferson F. (2019). A Comparative Analysis of Student Performance in an Online vs. Face-to-Face Environmental Science Course from 2009 to 2016. Front. Comput. Sci..

[12] Sela Y., Grinberg K., Shapiro Y., Nissanholtz-Gannot R. (2020). A cross-sectional study on preferred employment settings of final-year nursing students in Israel. Hum. Resour. Health.

[13] Hiromi K., Rahman M., Mika I., Chieko K. (2020). Effectiveness of a Basic Education Program on Radiation Related Health Concerns for Nurses of Public Health and School Health in Japan. Iran. J. Public Health.

[14] Kawasaki H., Yamasaki S., Masuoka Y., Iwasa M., Fukita S., Matsuyama R. (2021). Remote Teaching Due to COVID-19: An Exploration of Its Effectiveness and Issues. Int. J. Environ. Res. Public Health.

[15] Sphere Association (2018). The Sphere Handbook: Humanitarian Charter and Minimum Standards in Humanitarian Response.

[16] Schretlen D.J., Munro C.A., Anthony J.C., Pearlson G.D. (2003). Examining the range of normal intraindividual variability in neuropsychological test performance. J. Int. Neuropsychol. Soc..

[17] Siegelman N., Bogaerts L., Frost R. (2017). Measuring individual differences in statistical learning: Current pitfalls and possible solutions. Behav. Res. Methods.

[18] R Core Team (2020). R: A Language and Environment for Statistical Computing.

[19] Murre J.M.J., Dros J. (2015). Replication and Analysis of Ebbinghaus’ Forgetting Curve. PLoS ONE.

[20] Maniago J.D., Feliciano E.E., Santos A.M., Agunod C.L., Adolfo C.S., Vasquez B.A., Albougami A., Almazan J.U. (2021). Barriers in performing physical assessment among nursing students: An integrative review. Int. J. Nurs. Sci..

[21] Jung C.-C., Chen N.-T., Hsia Y.-F., Hsu N.-Y., Su H.-J. (2021). Influence of Indoor Temperature Exposure on Emergency Department Visits Due to Infectious and Non-Infectious Respiratory Diseases for Older People. Int. J. Environ. Res. Public Health.

[22] Kim J.S., Gu M.O., Chang H. (2019). Effects of an evidence-based practice education program using multifaceted interventions: A quasi-experimental study with undergraduate nursing students. BMC Med. Educ..

[23] Tlali T.V., Baliyan S.P. (2021). Gender, Age and Faculty Differences in Learning Practices among Undergraduates at the National University of Lesotho: Way Forward to Improve Learning. Creat. Educ..

[24] Ashcroft T.J., Lutfiyya Z.M. (2013). Nursing educators’ perspectives of students with disabilities: A grounded theory study. Nurse Educ. Today.

